# The Impact of Multiple Malignancies on Patients with Bladder Carcinoma: A Population-Based Study Using the SEER Database

**DOI:** 10.1155/2009/406965

**Published:** 2009-12-28

**Authors:** Joshua R. Ehrlich, Michael J. Schwartz, Casey K. Ng, Eric C. Kauffman, Douglas S. Scherr

**Affiliations:** Department of Urology, New York Presbyterian Hospital, Weill Medical College of Cornell University, 525 East 68th Street, Starr 900, New York, NY 10021, USA

## Abstract

*Purpose*. To date, no study has examined a population-based registry to determine the impact of multiple malignancies on survival of bladder cancer patients. Our experience suggests that bladder cancer patients with multiple malignancies may have relatively positive outcomes. *Materials & Methods*. We utilized data from the Surveillance Epidemiology and End Results (SEERs) database to examine survival between patients with only bladder cancer (BO) and with bladder cancer and additional cancer(s) antecedent (AB), subsequent (BS), or antecedent and subsequent to bladder cancer (ABS). *Results*. Analyses demonstrated diminished survival among AB and ABS cohorts. However, when cohorts were substratified by stage, patients in the high-stage BS cohort appeared to have a survival advantage over high-stage BO patients. *Conclusions*. Bladder cancer patients with multiple malignancies have diminished survival. The survival advantage of high-stage BS patients is likely a statistical phenomenon. Such findings are important to shape future research and to improve our understanding of patients with multiple malignancies.

## 1. Introduction

Patients with cancer are at an increased risk for developing additional subsequent primary tumors [1]. One recent study using Surveillance Epidemiology and End Results (SEERs) data found that of cancer patients alive as of January 1, 2001, nearly 8% were diagnosed with more than one primary malignant tumor between 1975 and 2001 [2]. This statistic underscores the pervasive nature of multiple malignancies and raises important questions regarding etiology, treatment decisions, demographics, and outcomes. 

 While several investigations have focused on assessing risk factors for developing subsequent primary malignancies, long-term outcomes data on patients with multiple malignancies are lacking. Specifically, to our knowledge, no study has examined a large cohort of bladder cancer patients to compare survival between those with single and multiple cancers. Based on our own clinical experience, we hypothesized that patients with bladder cancer and multiple malignancies may have improved outcomes compared with their single malignancy counterparts. Aside from clinical observations, this hypothesis stemmed, in part, from the question: if a patient survives one malignancy, is there perhaps something inherently different in their immune system or other genetic surveillance mechanisms that might confer improved survival? 

 Bladder cancer is, in fact, an immunoresponsive malignancy. Numerous studies have demonstrated the efficacy of bacillus Calmette-Guerin immunotherapy in eradicating bladder tumors and delaying recurrence and stage progression of noninvasive bladder cancer [3, 4]. While not completely understood, the underlying mechanism seems to involve a vigorous cellular immune response with the interaction of sensitized T-lymphocytes and activated macrophages through cytokine production to kill and prevent growth of cancer cells [5, 6]. Immune surveillance in bladder cancer patients with multiple malignancies may indeed be different from patients with only bladder cancer only, and such differences may manifest in population-based data relating to survival. An investigation comparing long-term survival among patients with single and multiple cancers might provide insight into the biological, genetic and immunological factors that influence the development of multiple cancers, treatment response, and cancer survival. In addition, it may provide an improved understanding of the impact of an aging population, increased cancer screening, and improved imaging techniques in detecting additional cancers. 

 Because our own cohort of bladder cancer patients with multiple malignancies is relatively small, we employed data from the SEER program database to investigate differences in survival from bladder cancer among patients with single and multiple malignancies. Survival among patients diagnosed with only bladder cancer was compared with patients diagnosed with bladder cancer and one or more additional subsequent or antecedent nonbladder malignancies. We compared these cohorts, adjusting for risk and demographic factors in order to determine trends in survival from bladder cancer.

## 2. Methods

All analyses were carried out using SEER data compiled from 1973 to 2004. The SEER Program collects cancer incidence data from population-based cancer registries that covered greater than 10% of the US population in 2000 [1]. A single malignancy cohort was defined to consist of all patients who received a diagnosis of bladder cancer and no additional cancer diagnoses (BO). The cohort of patients with both bladder and nonbladder malignancies was partitioned according to whether the nonbladder malignancy occurred antecedent (AB), subsequent (BS), or both antecedent and subsequent (ABS) to the diagnosis of bladder cancer (Table 1). All nonbladder malignancy sites tracked in SEER were included in the analyses (see: http://seer.cancer.gov/siterecode/icdo3_d01272003/); nonmelanoma skin cancers were not included. Native SEER patient coding was used to determine the number of cancers diagnosed in the same individual and cancer survival was followed based on individual, not population, records. All survival times were calculated from the date of diagnosis of the bladder cancer and were censored at 5- and 10-year endpoints. Patients were excluded from analysis if data were not available for any of the following variables: age, sex, race, grade, stage and year of diagnosis; additionally, due to the small number of patients not classified in SEER as white, black or Asian/Pacific Islander these patients were excluded. A total of 162,181 patients were included in the analysis, and 31,649 patients were excluded using these criteria. 

The univariate Kaplan-Meier (KM) method was used to estimate the survival rate and construct overall, cancer-specific and bladder cancer-specific survival curves with data censored at 5- and 10-year endpoints; a log-rank test was used to determine *P*-values for these data. A multivariate Cox proportional hazards model was used to calculate hazard ratios (HRs) of death for malignancy groups, adjusted for age, sex, race, grade, stage, and year of diagnosis in order to avoid confounding effects of these variables. Univariate and multivariate survival analyses were repeated with select cohorts further partitioned based on the staging of their bladder cancer as “low” (localized) or “high” (regional or distant), according to the SEER Summary Staging classification (Table 1). Cause of death, as coded in SEER, was used in calculating cancer-specific and bladder cancer-specific survivals. SAS 9.1 (SAS Institutes, Cary, NC) was used for all statistical analyses. 

 The mean time between cancer diagnoses for the AB and BS, cohorts was determined and compared using a two-sided *t*-test; the same procedure was conducted to determine the mean time between the diagnosis of bladder cancer and death for the BO, AB, BS and ABS groups. Lastly, SEER patient records were counted to determine the most common nonbladder malignancies overall and in each multiple malignancy cohort. All statistical tests were two-sided, with a 0.05 level for statistical significance.

## 3. Results

**Figure 1:**
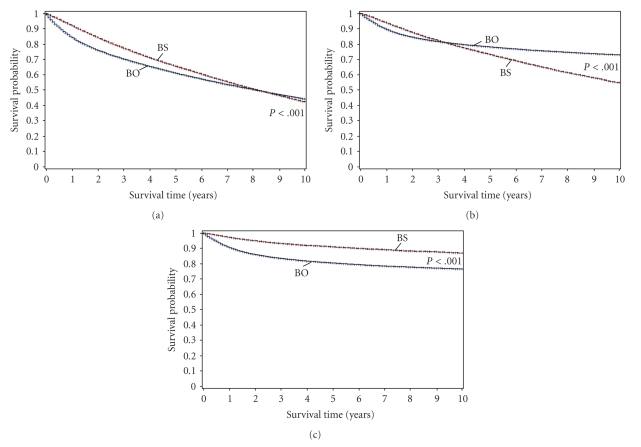
Kaplan-Meier curves comparing (a) overall, (b) cancer-specific and (c) bladder cancer-specific survival of bladder cancer only and BS patients with data censored at a 10-year endpoint.

KM curves and univariate analyses show that the BO group has an overall survival advantage at 5- and 10 years against all multiple malignancy cohorts except for the BS group (Figure 1, Table 2). Our finding that the BS group had the greatest overall survival advantage at 5 years and yet the BO group had this advantage at the 10-year endpoint led us to construct a Cox proportional hazards model in order to more effectively analyze survival controlling for age, sex, race, grade, stage, and year of diagnosis. Multivariate analysis confirmed that BO patients have an overall and cancer-specific survival advantage at 5- and 10 years over the AB and ABS groups, but not over BS patients; BS patients had an increased overall and decreased cancer-specific survival compared to the BO group (Table 3). 

**Figure 2:**
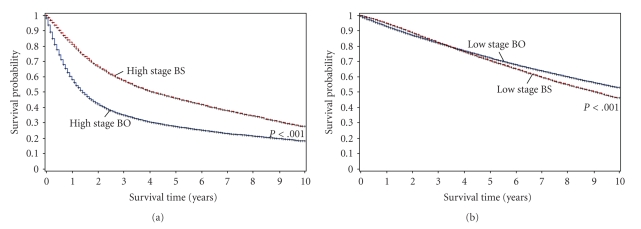
(a) Kaplan-Meier curves illustrate an overall survival advantage at 10 years for the high-stage BS cohort when compared to its respective high-stage bladder cancer only reference group. (b) The opposite finding existed for the low-stage patient groups, where the bladder cancer only patients had a slightly increased survival.

We then partitioned BS and BO patients based on the staging of their bladder cancer, according to the SEER summary staging schema, and analyzed this data using a multivariate Cox proportional hazards model. Our analyses demonstrated a notable increase in survival among BS patients with high-stage bladder cancer as compared to high-stage BO patients, while the opposite finding existed for the low-stage cohorts where BO patients had a modest survival advantage (Table 4, Figure 2). All survival differences were statistically significant with a *P*-value <.001 (Tables 3-4). Thus, it appears that BO patients with bladder cancer and no other malignancies have a survival advantage over most patients with multiple cancers. However, even after adjusting for key differences between subgroups, patients who develop at least one malignancy subsequent to the diagnosis of high-stage bladder cancer have an improved survival compared to patients with only high-stage bladder cancer. 

In order to better understand survival trends in our data we analyzed the time between diagnosis of bladder and nonbladder malignancies for the AB and BS groups, as well as the mean time to death following bladder cancer diagnosis in all groups. For both of these measures we demonstrated a significant difference between all groups, with BS patients having the longest time to death from the diagnosis of bladder cancer and high-stage BS patients having the shortest time between cancer diagnoses (Table 5).

Additionally, we sought to determine the most common nonbladder malignancies in patients with bladder cancer. We evaluated data for the overall study population and with patients partitioned by individual cohort in order to investigate differences in patterns of carcinogenesis and cancer diagnosis that might influence survival. The most common nonbladder tumor in each of the three multiple malignancy groups were prostate, followed by kidney in the AB group and lung cancer in the BS and ABS groups (Table 6).

## 4. Discussion

This study examines a population-based cancer registry, comparing survival among bladder cancer patients with single and multiple malignancies. Using SEER data collected from 1973–2004, we determined that AB and ABS patients have a distinct survival disadvantage when compared to BO patients. Several factors may account for this finding. First, patients with antecedent cancers who develop bladder cancer may have inherent deficiencies in immune surveillance, DNA repair, or epigenetic mechanisms leading to the development of multiple malignancies [7]. Similar acquired defects may also play a role in tumorgenesis in patients with previous cancer diagnoses [8, 9]. Moreover, the development of these deficiencies may be accelerated by smoking, obesity, and/or treatment burden (chemotherapy or radiation) associated with antecedent malignancies. Several well-known examples illustrate this point. 

 Cyclophosphamide has a clear association with bladder cancer—it is for this reason that urologists perform bladder cancer screening in this population [10]. In addition, patients who receive radiation for the treatment of prostate cancer are more likely to be diagnosed with bladder cancer, and more likely to develop high-grade disease and have diminished survival [11–13]. The development of subsequent cancer of the bladder and other sites is seen predominantly among patients who have undergone external beam radiation, not brachytherapy [14]. These observations may partially account for the negatively affected survival among the AB and ABS cohorts in this study. Of note, the most common nonbladder malignancy in all multiple malignancy groups was prostate cancer. Many patients in the BS group likely had their prostate removed at the time of cystectomy. We suspect that many of the prostate cancers diagnosed in this group may have been found incidentally upon review of surgical specimens. This may explain the high incidence of subsequently diagnosed prostate cancers among a group where many have had their prostate removed. Future work might employ SEER-Medicare data to examine the correlation between prostate radiation and bladder carcinoma and differential survival among patients with both these malignancies. 

 Aside from acquired genetic or immune surveillance deficiencies, treatment burden from previous cancers may also predispose patients to noncancer related comorbidities such as chronic renal insufficiency and cardiovascular disease, leading to decreased survival [15, 16]. These comorbidities may also preclude the use of optimal cancer therapies in patients with antecedent cancers, as they may be unable or unwilling to undergo recommended treatments. This factor almost certainly contributes to our observation of diminished survival in the AB and ABS patient cohorts. Although seemingly intuitive, these findings are important to document because they can and should play a role in clinical practice. Treatment decisions may be significantly influenced by such information, and counseling a patient with only bladder cancer is quite different than a patient with bladder cancer and an antecedent malignancy. 

 Contrary to our observations in the AB and ABS cohorts, we observed that patients who develop a malignancy subsequent to high-stage bladder cancer had improved overall, cancer-specific and bladder cancer-specific survival when compared to patients with only high-stage bladder cancer. Although this specific finding supports our initial hypothesis, we believe it is more likely the result of statistical and not biological phenomena. The BS group has the longest mean survival from the time of diagnosis of bladder cancer of any group analyzed. This finding raises the possibility that the reason for the BS group's apparent survival advantage is that the only patients included in the BS cohort are those who live long enough to develop another cancer following their bladder cancer diagnosis. This effect is specifically seen amongst high-stage cancer patients since they are most likely to die of their disease; those receiving a second cancer diagnosis may become part of the BS group since they have outlived other high-stage bladder cancer patients, not because their second malignancy confers a protective effect. While it is conceivable that a true biological phenomenon is responsible for improved survival in some patient cohorts with an increased cancer burden, this appears unlikely and is not possible to fully determine given our data. 

 However, our study is not the first to speculate on improved outcomes among patients multiple malignancies patients. Duchateau and Stokkel examined the prevalence of multiple malignancies among patients with nonsmall cell lung cancer (NSCLC) and compared survival between patients with only NSCLC and various cohorts with NSCLC and additional cancers [17]. They found that patients with only NSCLC had the least favorable outcome, whereas those with at least two cancers in addition to a primary diagnosis of NSCLC had the greatest survival, and patients with one cancer in addition to a primary NSCLC diagnosis had an intermediate outcome. One weakness of this study may be that approximately 25% of patients had multiple NSCLC tumors that were included in the analysis as separately diagnosed malignancies. The authors note that TNM stage and treatment were comparable between all groups, and they speculate that tumors in patients with multiple malignancies may actually have distinct growth habits that are responsible for the finding in this study. 

 Our analysis sought to control for a number of important confounding variables, including: age, sex, race, grade, stage, and year of diagnosis. It should be noted that while we used the reported cause of death in SEER for our analyses, it may be especially difficult to assign a single cause of death to a patient with multiple cancers. Also, since SEER records cancer diagnoses just as they are reported, it is possible that some reported second malignancies were not diagnosed histologically and were actually mestastatic lesions. Finally, we considered examining whether or not differences in treatment existed between cohorts; however data was inconsistent and not comprehensive within this SEER dataset (SEER-Medicare allows more complete access to treatment data), and so we chose not to include it as a variable. It would be useful to investigate possible treatment and practice pattern differences between these cohorts in a future study, as determining if such differences do exist may help elucidate reasons for variable outcome between cohorts. 

 The SEER Summary Staging classification, which differentiates low stage (local) and high-stage (regional or distant) disease, is an important limitation of this study. The American Joint Committee on Cancer (AJCC) method is more commonly used in clinical settings, while SEER has standardized and simplified staging to ensure consistent definitions over time. AJCC staging is available for more recent years through SEER, however, we chose to use the SEER Summary Staging classification because it was the most consistent over the entire time period examined (1973–2004). The limitation exists due to the variability of patients in the high- and low-stage groups. For example, “low-stage” encompasses noninvasive and localized invasive disease (Ta, Tis, T1, and localized T2) in the same cohort, even though the patient population within this cohort is heterogeneous, as are treatment strategies; this makes comparison between BO, AB, ABS, and BS cohorts by stage more difficult to interpret. Part of the explanation for the survival advantage among high-stage BS patients relative to high-stage BO patients may stem from this limitation. Comparisons between overall cohorts may also be affected by these groupings. Unfortunately, the extent of such an effect is hard to measure. Nevertheless, we believe that the sample size of each cohort is large enough that the observations of diminished survival among multiple malignancy patients are valid. 

 Finally, we included all bladder cancer reported in SEER as part of this investigation and did not differentiate urothelial carcinoma from other bladder malignancies. Given that urothelial carcinoma comprises a large proportion of bladder cancer (>90%), the effect on this study is likely minimal, but worthy of mention. Because cancers such as small cell carcinoma or squamous cell carcinoma of the bladder typically present at higher stages and carry worse prognoses, it is possible that these are disproportionately represented in the “high-stage” groupings, thereby negatively skewing survival data for these patients. Again, however, the proportion of bladder cancers represented by these rare diagnoses is small and unlikely to significantly affect the study outcome. 

 It should also be noted that epidemiological information regarding the differential survival of cancer cohorts may not be appropriate to directly inform individual patient care. However, these findings are important to shape future research, better understand multiple malignancy patients, and ultimately improve oncologic practice.

## 5. Conclusions

Bladder cancer patients with antecedent malignancies have diminished survival when compared to patients with only bladder cancer. An awareness and understanding of the differential survival between such defined cancer patient cohorts can help to inform and improve future oncologic research and our understanding of patients with multiple malignancies. Future survival analyses among more homogeneous multiple malignancy populations will be important in order to determine optimal treatment for their cancer(s) and evidence-based information regarding their prognosis.

## Figures and Tables

**Table 1:** 

Cohort definitions	
BO	Bladder cancer only: patients diagnosed with bladder cancer and no other cancers
AB	Antecedent cancer(s) + bladder cancer: patients diagnosed with nonbladder malignancy before being diagnosed with bladder cancer
BS	Bladder cancer + subsequent cancer(s): patients diagnosed with nonbladder malignancy after being diagnosed with bladder cancer
ABS	Antecedent cancer + bladder cancer + subsequent cancer: patients diagnosed with nonbladder malignancy both before and after being diagnosed with bladder cancer

SEER Summary Staging Definitions	

Low-stage	Localized disease
High-stage	Regional or distant disease

Variables adjusted for in Cox model	

Age	Grade
Sex	Stage
Race	Year of diagnosis

**Table 2:** Survival rates for single and multiple malignancy cohorts, univariate analysis.

		5-yr overall	10-yr overall	5-yr	10-yr	5-yr bladder	10-yr bladder
				CA specific	CA specific	CA specific	CA specific
BO *n* = 118,101	survival rate	0.61	0.44	0.78	0.73	0.80	0.76
AB *n* = 17,200	survival rate	0.52	0.27	0.61	0.39	0.91	0.86
	*P*-value	<.001	<.001	<.001	<.001	<.001	<.001
ABS *n* = 3,141	survival rate	0.46	0.26	0.62	0.52	0.82	0.77
	*P*-value	<.001	<.001	<.001	<.001	<.001	<.001
BS *n* = 23,739	survival rate	0.66	0.43	0.74	0.55	0.91	0.87
	*P*-value	<.001	<.001	<.001	<.001	<.001	<.001

*P*-values were all calculated against the corresponding survival rate of the bladder cancer only group. CA : cancer.

**Table 3:** Survival rates and Cox hazard ratios representing AB, BS, ABS, and BO cohorts, multivariate analysis.

		5-yr overall	10-yr overall	5-yr	10-yr	5-yr bladder	10-yr bladder
				CA specific	CA specific	CA specific	CA specific
AB (*n* = 17200)	HR	1.409	1.384	1.788	1.842	0.892	0.906
	95% CI	(1.375, 1.444)	(1.353, 1.416)	(1.733, 1.845)	(1.788, 1.898)	(0.852, 0.933)	(0.867, 0.947)
BS (*n* = 23739)	HR	0.824	0.936	1.191	1.522	0.446	0.488
	95% CI	(0.804, 0.845)	(0.917, 0.955)	(1.156, 1.228)	(1.484, 1.561)	(0.424, 0.468)	(0.467, 0.510)
ABS (*n* = 3141)	HR	1.163	1.255	1.882	2.209	0.485	0.517
	95% CI	(1.102, 1.227)	(1.198, 1.314)	(1.769, 2.002)	(2.093, 2.331)	(0.425, 0.552)	(0.458, 0.583)
BO (*n* = 118101)	HR	1.0	1.0	1.0	1.0	1.0	1.0

HRs reported with 95% confidence intervals (95% CI). All HRs calculated using BO cohort as the reference group. CA : cancer.

**Table 4:** Stage adjusted survival rates and Cox hazard ratios for BO and BS cohorts, multivariate analysis.

		5-yr overall	10-yr overall	5-yr	10-yr	5-yr bladder	10-yr bladder
				CA specific	CA specific	CA specific	CA specific
BO, low stage (*n* = 81, 129)	survival rate	0.73	0.53	0.90	0.85	0.92	0.88
	HR	0.519	0.562	0.255	0.252	0.423	0.462
	95% CI	(0.497, 0.542)	(0.540, 0.584)	(0.242, 0.269)	(0.240, 0.264)	(0.396, 0.452)	(0.433, 0.492)
BS, low stage (*n* = 18, 778)	survival rate	0.71	0.46	0.78	0.59	0.96	0.92
	HR	0.537	0.642	0.596	0.738	0.223	0.285
	95% CI	(0.511, 0.564)	(0.616, 0.670)	(0.563, 0.631)	(0.702, 0.775)	(0.202, 0.245)	(0.262, 0.311)
BO, high-stage (*n* = 28, 972)	survival rate	0.28	0.18	0.39	0.33	0.43	0.38
	HR	1.802	1.682	1.700	1.546	2.644	2.586
	95% CI	(1.727, 1.880)	(1.619, 1.748)	(1.620, 1.783)	(1.479, 1.615)	(2.486, 2.813)	(2.438, 2.744)
BS, high-stage (*n* = 4, 961)	survival rate	0.46	0.27	0.54	0.39	0.72	0.65

HRs reported with 95% confidence intervals (95% CI). All HRs were calculated using the high-stage BS cohort as the reference group. SEER staging classification.

**Table:** (a) Time between bladder and nonbladder cancer diagnoses

Patient cohort		Mean time between diagnoses ± SD (months)
AB	Overall	51.00 ± 61.80
	Low stage only	48.60 ± 60.48
	High-stage only	59.52 ± 65.64
BS	Overall	56.28 ± 63.48
	Low stage only	63.36 ± 65.88
	High-stage only	29.16 ± 51.36

*P* < .001 for all intergroup comparisons.

**Table:** (b) Time to death following diagnosis of bladder cancer

Patient cohort	Mean time to death following
	bladder cancer diagnosis (months)
BO	64.30
AB	43.21
BS	91.80
ABS	62.80

*P* < .001 for all intergroup comparisons.

**Table 6:** Most common nonbladder cancer diagnoses.

Overall (no. patients)	AB	BS	ABS
(1) Prostate (16,160)	(1) Prostate (6,568)	(1) Prostate (8,214)	(1) Prostate (1,378)
(2) Lung (6,992)	(2) Renal (1,926)	(2) Lung (4,912)	(2) Lung (871)
(3) Renal (4,467)	(3) Colon (1,646)	(3) Renal (1,887)	(3) Renal (654)
(4) Colon (4,061)	(4) Breast (1,421)	(4) Colon (1,855)	(5) Colon (560)
(5) Breast (2,627)	(5) Lung (1,209)	(5) Breast (929)	
